# Effects of dietary supplementation with different levels and molecular weights of fungal β-glucan on performances, health and meat quality in broilers

**DOI:** 10.5713/ajas.18.0927

**Published:** 2019-03-07

**Authors:** Attawit Kovitvadhi, Pipatpong Chundang, Chanin Tirawattanawanich, Wai Prathumpai, Pawadee Methacanon, Krith Chokpipatpol

**Affiliations:** 1 Department of Physiology, Faculty of Veterinary Medicine, Kasetsart University, Bangkok 10900, Thailand; 2 Innovation Cluster 2, Thailand Science Park, Ministry of Science and Technology, Pathum Thani 12120, Thailand; 3 Microbial Biotechnology and Biochemicals Research Unit, National Center for Genetic Engineering and Biotechnology, National Science and Technology Development Agency, Pathum Thani 12120, Thailand; 4 National Metal and Materials Technology Center (MTEC), National Science and Technology Development Agency, Pathum Thani 12120, Thailand; 5 Asia Star Trade Co., Ltd., Bangkok, 10400, Thailand

**Keywords:** Amylase, Bursa of Fabricius, Digestive Enzyme Activity, Immunity, *Ophiocordyceps dipterigena*, Phagocytosis

## Abstract

**Objective:**

To investigate the effects of dietary supplementation with different levels and molecular weights of fungal β-glucan on productive performances, health, carcass traits and meat quality in broilers.

**Methods:**

Two hundred and ten of one-day-old chicks with equal sex were assigned to seven experimental groups in 2×4 factorial arrangement. These groups were supplemented with (0, 10, 30, and 60 ppm) of molecular weight 1–3, 1–6 β-glucan (low or high). High molecular weight β-glucan (H: 943 kDa) was obtained from *Ophiocordyceps dipterigena* BCC 2073, whereas H with γ-Irradiation treatment was performed to achieve low molecular weight β-glucan (L: 8 kDa).

**Results:**

There was no statistical significance in productive performances, apparent digestibility and interaction between fixed factors along 42 days of experiment (p>0.05). A higher caecal amylase activity was present in the group that received L, while there was a dramatic decrease in H and the control groups, respectively (p<0.05). The increase of supplemental dose increased caecal amylase activity (p<0.05). Immunomodulatory effects from L was revealed by the marked increase of phagocytic activity, relative weight of thymus and bursa of fabricius (p<0.05). Similarly, the additive dose at 30 ppm provided the same results, whereas the only significant difference with supplementation at 60 ppm was an increase in phagocytic activity (p<0.05). Interestingly, villi height of broilers fed L was higher than other groups (p<0.05). The treatments did not influence haematology, blood chemistry, antibody production level against vaccination, carcass traits and meat quality (p>0.05).

**Conclusion:**

The supplementation of L at 30 ppm was suggested to achieve benefits of immune modulation without adverse effects on other parameters.

## INTRODUCTION

The ban of using antibiotics as growth promoters was announced in several countries to reduce the problems of bacterial resistance and antibiotic residue in animal products. A deterioration of animals’ performance, health and products were observed after this regulation [1]. Therefore, several techniques have been studied to solve this problem. Researchers have studied dietary supplements with growth promoters and immunomodulation properties in an attempt to solve this problem.

β-Glucan is non-starch polysaccharide which occurs in cell structure of cereal crops (mainly barley and oat), yeasts, mushrooms and molds [1–3]. The diverse chemical structure of β-glucan is reported as depending on the origin which influences the physiological response after the usage. β-(1→3)-linkages are the basic structure of β-glucan, whereas β-(1→4)-linkages and β-(1→6)-linkages are found only in plants and fungi, respectively [3]. The intake of (1→3) and (1→4)-β-glucans from plants have negative effects because these structures are insoluble and gel forming with the consequence of lower nutrient digestibility in broilers and atrophy of intestinal villi [1,4,5]. On the other hand, several benefits to broilers such as growth performances, immune-modulatory properties and meat quality [6–20] were revealed after feeding supplements with (1→3) and (1→6)-β-glucans from yeast, bacteria or mold. Moreover, the mortality rates of broilers after pathogen challenge test were lower in the group with β-glucans supplementation comparing to the control group fed basal diet without the supplements [10,11,13–15,19,20]. Therefore, β-glucans were considered as immunomodulator supplements in broilers [1] and humans [3]. The research to date has tended to focus on using yeast β-glucans rather than mold (*Ophiocordyceps dipterigena*) which has a similar structure [17]. Interestingly, low molecular weight (5 kDa) β-glucans exhibited the highest function to induce interleukin-8 production comparing to the β-glucans with higher molecular weight [2]. Therefore, the using β-glucans with low molecular weight in broilers should provide better benefits than using β-glucans with high molecular weight.

The objective of this study was to determine the consequences of using different levels and molecular weights of fungal β-glucan as dietary supplementation on productive performances, health, carcass traits and meat quality in broiler chickens.

## MATERIALS AND METHODS

### Fungal β-glucan preparation

*Ophiocordyceps dipterigena* BCC 2073 was grown initially on potato dextrose agar (Difco, Becton, Dickinson and Company, Franklin Lakes, NJ, USA) at 25°C for 5 to 7 days. An agar block (1 cm^3^) containing the growing culture was cut into small pieces and transferred to 25 mL of potato dextrose broth (Difco, Becton, Dickinson and company, USA) in a 250-mL Erlenmeyer flask. This liquid seed culture was incubated for 5 to 7 days at 25°C on a rotary shaker at a shaking speed of 200 rpm (New Brunswick, Franklin Lakes, NJ, USA). The medium used in a 300-L fermenter consisted of 2.5 g/L yeast extract, 68.98 g/L hydrolyzed cassava starch, 2.62 g/L molasses, 0.5 g/L KH_2_PO_4_, 0.2 g/L K_2_HPO_4_, 0.2 g/L MgSO_4_ ·7H_2_O, 0.14 g/L MnSO_4_·H_2_O, 1 mL/L trace element solution (trace elements consisted of 14.3 g/L ZnSO_4_·H_2_O, 2.5 g/L CuSO_4_·5H_2_O, 0.5 g/L NiCl_2_·6H_2_O and 13.8 g/L FeSO_4_·H_2_O) and 1 mL/L vitamin solution (Blackmores, Warriewood, NSW, Australia). The culture was agitated at 120 to 180 rpm and aerated at 1 vvm, but pH was not controlled. The cultivation was carried out for 10 days. The culture filtrate was then mixed with four volumes of 95% ethanol, stirred vigorously for 10 to 15 min and stored at 20°C for at least 12 h. β-Glucan was redissolved in distilled water, and any insoluble material was removed by centrifugation at 10,000 *g* for 20 min. The supernatant was then dialyzed (2 kDa molecular weight cut off, Spectrum Laboratories, Rancho Dominguez, CA, USA) against 4 L of distilled water for 24 h and drum dried to achieve high molecular weight β-glucan (943 kDa). γ-Irradiation of the glucan solution was carried out using a cobalt-60 irradiator (Gammacell 220 Excel, MDS Nordion, Ottawa, Canada). The doses applied in this work were 50 kGy at room temperature to obtain the β-glucan size of 8 kDa (low molecular weight). Dosimetry was carried out using 10×30×3 mm of Harwell Red Perspex 4034 (Harwell Dosimeters Ltd., Oxford, UK) calibrated against reference standard dosimeter (Fricke dosimeter) that was traceable to international standard set by the Office of Atoms for Peace, Bangkok, Thailand. The radiated β-glucan solution was then drum dried and stocked for dietary supplement experiments.

### Diets, animals, and housing

The basal diets for growing (1 to 21 days old) and finishing period (22 to 42 days old) met the nutrient requirements of Bangkok animal research centre Co., LTD (Bangkok, Thailand). The ingredients and chemical composition of experimental diets are illustrated in Table 1. The β-glucan samples were provided by Asia Star Trade Co., LTD (Bangkok, Thailand) and BIOTEC with purity at 94.5% and 92.5% dry matter (DM) for low and high molecular weight, respectively.

The complete randomized block design was performed in 2×4 factorial arrangement with molecular weight of β-glucan (low or high) and supplemental doses (0, 10, 30, and 60 ppm) as main effects. Two hundred and ten of one-day-old chicks of equal sex from a commercial hatchery (Avian CP, Charoen Pokphand Foods PCL., Bangkok, Thailand) were transported to the Animal Experimental Unit (Faculty of Veterinary Medicine, Kasetsart University, Bangkok, Thailand). The chicks were randomly assigned into 7 experimental groups as follows: i) control group fed the basal diet (C), basal diet with; ii) low dose (10 ppm); iii) medium dose (30 ppm); iv) and high dose (60 ppm) of low molecular weight β-glucan; v) basal diet with low dose (10 ppm); vi) medium dose (30 ppm); vii) and high dose (60 ppm) of high molecular weight β-glucan. Each treatment group contain 5 replicate pens and 6 birds per pen.

All broilers had free access feed and clean water (*ad libitum*), aand were kept in a closed housing system with 18 L:6 D lighting program. The temperature in the room was controlled at 34°C for 5 days and then gradually decreased at 2°C per week to reach 26°C which was maintained until the end of the experiments (42 days). Two broilers from each pen were euthanasia by intravenous injection of pentobarbital sodium (100 mg/kg body weight) at 25 and 32 days of age to collect the samples for evaluation on health parameters and phagocytic activity, respectively. This experiment was approved by the Institutional Animal Care and Use Committee of Kasetsart University, Bangkok, Thailand (ACKU60-VET-026).

### Growth performances and apparent digestibility

The live weight and feed intake were checked every two weeks from 1 to 42 days old to determine average daily feed intake (ADFI), average daily weight gain (ADG) and feed conversion ratio (FCR). Mortality and morbidity were recorded daily by the same observer. For digestibility study, the feed intake and amount of excreta was collected from 28 to 32 days of age. The diets and excreta were analyzed in duplicate for DM, crude protein (CP), ether extract (EE), and crude ash by ignition to 550°C for organic matter (OM) calculation, according to the Association of Official Analytical Chemists [21]. The uric acid content in excreta was analysed to correct the CP digestibility [22].

### Health parameters

Two broilers of from pen were euthanized at 25 days of age to study health parameters. Pancreas, spleen, thymus and bursa of fabricius were weighted and presented as percentage of live weight. Blood haematology and blood chemistry were analyzed at Kasetsart University Veterinary Teaching Hospital, Kasetsart University (Kamphaeng Saen campus). The mid-duodenum of euthanized broilers was immerged in 10% buffered formalin for 30 days and then the tissue samples were processed, embedded in paraffin, sectioned at 5-μm thicknesses by a rotary microtome (Leica RM2155; Leica Instruments GmbH, Nussloch, Germany) and stained with haematoxylin and eosin. Villi height and crypt depth were measured under a microscope using an image analysis programme (Image Pro Plus; Media Cybernetics, Bethesda, MD, USA). All broilers were nasal vaccinated by live attenuated New Castle disease virus (NDV: Bio-Vac LS-H120, Fatro S.p.A., Bologna, Italy) at 5 and 18 days of age. The serum samples were collected at 25 days of age to determine antibody titer against NDV by hemagglutination inhibition assay.

### Abdominal exudative cell phagocytic activity

Before the beginning of experimental, antibody against sheep red blood cell (sRBC) was prepared by intravenous injection with 1 mL of 7% (v/v) sRBC into two adult chickens at 6 and 7 weeks of age. Then, the sera with anti-sRBC antibody were collected and kept at −20°C at 14 days after the last injection for sRBC opsonization in a further step. One broiler from each pen was randomly selected to study abdominal exudative cell (AEC) phagocytic activity. The selected chicken at 22 days old was administered intra-abdominally at 1 mL/100 g body weight of 3% (w/v) sephadex G-50 (Merck, Kenilworth, NJ, USA) in phosphate-buffered saline (PBS, pH 7.4). At 72 h post-injection, the birds were euthanized, the abdominal cavity opened and flushed three times with 10 mL each of cold sterile PBS (pH 7.4) to harvest abdominal fluid in a sterile tube that was placed on ice for 20 min to allow sedimentation of tissue debris. The fluid was aspirated and centrifuged at 400 g for 10 min (4°C) to collect the pelleted AEC which then washed three times by PBS (pH 7.4). The AEC was reconstituted to 1×10^5^ cells/mL by minimum essential medium with 5% (v/v) fetal bovine serum.

The freshly sRBC in Alsever’s solution were washed 3 times in sterile PBS (pH 7.4), reconstituted to a 5% (v/v) concentration in PBS (pH 7.4), and opsonized by incubation with a subagglutinating concentration of heat-inactivated chicken anti-sRBC serum for 30 minutes at room temperature. After the incubation, the opsonized sRBC were washed in sterile PBS (pH 7.4) and reconstituted as a 5% (v/v) suspension in complete medium. A suspension of 5% (v/v) unopsonized sRBC was prepared in the same previous protocol except without incubation with the anti-sRBC serum. A 100-μL drop of the AEC suspension on a coverslip was prepared in 2 sets of triplicate coverslips. The coverslips were left at room temperature for 30 min to allow cell-surface adhesion. Unattached cells were removed by washing three times by PBS (pH 7.4). One set of coverslips was overlaid with the 5% (v/v) opsonized sRBC, and the other set was overlaid with the unopsonized sRBC. All samples were incubated at 38°C for 2 hours in a moisture chamber. Then, the coverslips were washed three times in PBS (pH 7.4), fixed in absolute methanol, stained with Wright’s stain and mounted. Percentage of phagocytic macrophages and amounts of phagocytosed sRBC per macrophage were counted for the calculation from 100 cells per replication.

### Digestive enzyme activities

All remaining 42 days old broilers were slaughtered to analyse gut enzyme activity, carcass traits and meat quality. For gut enzyme activities, the duodenal and caecal contents were collected immediately after the evisceration and stored in −20°C until analysis. The content samples of each organ (5 g) were subjected for homogenization with PBS (pH7, 1:2 w/v) on ice followed by centrifugation at 18,000 *g* for 30 minutes at 4°C. The supernatant containing a crude enzyme extract (CEX) was stored in −80°C for further analysis. Protease and amylase activity were evaluated in all content samples, whereas cellulase activity was studied only in the caecal content.

For protease activity, a set of equal volumes of substrate (2% casein in 0.1 N NaOH), CEX from duodenal or caecal content was mixed with 0.2 M PBS (pH 10) and then incubated at 4°C for 5 minutes. After the incubation, trichloroacetic acid was added to a final concentration of 5% v/v to stop the enzyme reaction before centrifugation at 5,000 g for 20 minutes at 40°C. The supernatant was mixed with 0.5 N NaOH and Folin-Ciocalteu reagent for further spectrophotometric absorbance measurement of free amino acids at a wavelength of 720 nm. *L*-tyrosine standard curve was used as a reference.

Soluble starch (5% w/v) was used as a substrate for determination of amylase activity of the CEX from duodenal or caecal content in 0.2 M PBS (pH 5). The mixture was incubated at 40°C and then 3,5-dinitrosalicylic acid (DNS) was added to final concentration of 1% v/v followed by 5-minute boiling at 100°C, then transferred to room temperature. Distilled water was added, and the absorbance was measured at 540 nm with maltose used to generate a standard curve.

The cellulase activity was assayed using 2% carboxyl methyl cellulose (CMC; low viscosity). The 75 μL of CMC was mixed with 425 μL of 0.2 M PBS (pH 8) and 50 μL of CEX from caecal content. The mixture was incubated at 37°C for 30 minutes, mixed with 250 μL of 1% DNS, and then boiled at 100°C for 10 minutes. After adding 2.5 mL of distilled water, the absorbance was measured at 540 nm against linear range of standard glucose. The absorbance in the formulation was deducted by a blank which contained all reagents but without the enzyme.

### Carcass traits and meat quality

Two broilers per pen (42 days old) were slaughtered at commercial abattoir to study carcass traits and meat quality. Carcass traits were recorded and then chilled in 4°C for 24 hours. Cold carcass weight (CCW) was determined after the chilling, whereas breast, wings, thighs and organs were dissected, and the proportion calculated. The meat bone ratio was evaluated from the proportion of meat and bone of the left thigh. The right pectoral muscle was collected to study pH and drip loss after 24 and 48 hours of chilling, respectively. The colour and chemical composition of the freeze-dried meat (moisture, CP, EE, and crude ash) were determined in left pectoral muscles. The thiobarbituric acid reactive substances assay was performed on the left breast meat after the storage for 1, 5, 7, 15, and 30 days at 4°C to determine lipid oxidation. Analyses were performed in duplicate and the results were expressed as μg malonyldialdehyde per kilogram of fresh meat, using a standard curve that covered a concentration range from 0.5 to 10 μM 1,1,3,3-tetramethoxypropane (Sigma-Aldrich, Steinheim, Germany). The absorbance was measured at 532 nm.

### Statistical analysis

The statistical analysis was performed by R-statistic, using Rcmdr Package in Rstudio [23]. With respect to the experimental design, a two-way analysis of variance was performed which molecular weight of β-glucan (low or high) and supplemental doses (0, 10, 30, and 60 ppm) were used as fixed effects. The interaction between these factors was evaluated. If a statistically significant difference was found, the different between factors was evaluated by Duncan’s new multiple range test. Statistical significance was accepted at p<0.05.

## RESULTS

### Productive performances, apparent digestibility, and gut enzyme activities

Productive performances during experiment are presented in Table 2, whereas apparent digestibility and gut enzyme activities are shown in Table 3. There were no statistical significances for live weight, ADG, ADFI, and FCR during the experiment. However, the ADG and ADFI declined when supplemented by L or H which contrasted to the dosage increment. The treatments did not influence apparent digestibility of DM, OM, CP, and EE (p>0.05). No morbidity and mortality were observed during experimental period. The increase of duodenal amylase activity was found in the supplemental group with L or H at 10 and 30 ppm but not at 60 ppm compared to the control group (p<0.05). The caecal amylase activity was higher in the treatments group which L provided the highest activity (p<0.05). Likewise, the supplementation at 10 and 30 ppm affected caecal amylase activity (p<0.05).

### Health parameters

Relative of organ weight at 25 days old, haematology, blood chemistry, phagocytic activity, antibody production and digestive tract histology of broilers are illusitrated as health parameters in Table 4. At 25 days of the experiment, the increase of relative weight of thymus and bursa of fabricius were observed in L group but not in control or H group (p< 0.05). In the same way, the supplementation at 30 ppm induced a higher relative weight of thymus and bursa of fabricius, while addition at 10 ppm only increased the weight of bursa of fabricius (p<0.05). No statistical difference was found in haematology (haematocrit, erythrocytes, and white blood cells) and blood chemistry (plasma protein, blood urea nitrogen, creatinine, alanine aminotransferase, cholesterol, and triglyceride) between the groups (p>0.05). The AECs had a greater efficiency to ingest opsonized sRBC than non-opsonized sRBC in all treatment groups. The phagocytic activities from broilers fed L were remarkably higher than other groups, which supplemental dose should be 30 or 60 ppm (p<0.05). Moreover, an increase of duodenal villi height was observed in the group provided with L in the diet (p< 0.05). Antibody titer against NDV from vaccination was not different between groups (p>0.05).

### Carcass traits and meat quality

The carcass characteristics and meat quality are presented in Table 5 and 6, respectively. The weight of spleen and gastrointestinal tract of broilers fed H were lower than other groups (p<0.05), whereas no statistical significance difference was observed for breast meat colour, pH24, drip loss percentage, chemical composition of meat and other carcass traits (p> 0.05). Lower lightness of *gastrocnemius* muscle was seen in the broilers’ meat which were fed H (p<0.05), while redness, yellowness, chroma and hue were not changed (p>0.05). The lower lipid oxidation was presented in the L group after storage at 4°C for 5 days comparing to other groups (p<0.05), however the supplements did not effect this parameter after storage for 1, 7, 15, and 30 days (p>0.05).

## DISCUSSION

### Productive performances, apparent digestibility, and gut enzyme activities

The dietary supplementation by β-glucan extracting from plants [5,18], mushroom (*Schizophyllum commune*; [7], mold (*Ophiocordyceps dipterigena* BCC 2073; [17]) and this study using mold did not improve productive performances. The positive outcomes in ADG and body weight were observed by adding β-glucan extracts from bacteria (*Agrobacterium* sp; [24]) and yeast (*Saccharomyces cerevisiae*; [8,12,16,25]). The increase of DM digestibility in treatment groups with yeast β-glucan should cause better performances [8], whereas this study did not find any difference in apparent digestibility. The yeast β-glucan supplementation at low level (140 ppm) did not improve productive performances [9], therefore the supplement should be around 200 to 2,000 ppm to obtain the benefits in ADG [8,25]. The long chain length, high molecular weight and (1→3)(1→4)-β-glucans structure of β-glucan from plants had a negative effect on ADG and FCR [4] resulting in lower digestibility efficiency from increased gut viscosity [3]. These adverse effects on performances were not reported after the using shorter chain, lower molecular weight and (1→3)(1→6)-β-glucans structure from yeast (around 800 kDa; [8,9,12,16,25]) and mold in this study (8 and 943 kDa for low and high molecular weight, respectively; [17]) as supplements. A decreasing trend without statistically significant differences in ADG and ADFI were observed in the treatment groups (Table 2) which may due to the immunomodulatory properties of β-glucan [6,7]. The surface topography of β-glucan is similar to the bacterial cell wall, therefore in healthy animals the immune response should respond after receptor recognition by immune cells [1]. The appropriate dose of β-glucan supplements can provide benefits in immunomodulation. On contrary, the lower productive performances were reported when the supplements were provided in excess concentration because most of digestible energy and protein were shifted to use in immune function rather than for growth [26]. The supplementation in 10, 30, and 60 ppm did not provide negative outcomes on FCR. Therefore, appropriate dosage of β-glucan must be studied to prevent the negative consequences for productive performances.

The enzyme activity extracted from duodenal and caecal content can be used as an indicator to represent the host’s digestive and bacterial activities, respectively. Obviously, amylase activity from caecal content was increased in the group which wert provided with L correlating to supplement concentration (Table 3). β-Glucans are non-digestible carbohydrates (prebiotics) by host enzymes but can be fermented by the intestinal microbiota. Indeed, *Lactobacillus* sp. (firmicutes) is present throughout the gastrointestinal tract (small intestinal and caecum) of poultry [27] as are other bacteria [28] which contain 1,3–1,4-β-glucanase activity. In the recent study, low molecular weight β-glucan in the chicken diet increased the activity of amylase. This result indicated that enzyme from microorganisms hydrolyzed β-glucan to glucose residues [28,29]. The results of these studies agreed with other findings which showed a higher amylase activity in duodenum and caecum in the supplement groups. The higher amylase activity refers to higher amount of amylolytic bacteria providing benefits of preventing pathogenic bacteria colonization in caecum by reduction pH and as well as competitive colonization [28]. Prathumpai et al [17] reported a higher *Lactobacillus* sp. and lower pathogenic bacteria after the supplements of fungal β-glucan in broilers. Molecular weight of β-glucan is an important factor to achieve these benefits because the higher amylase activity was observed in L compared to H. The smaller size and less complex structure are the benefits of low molecular weight β-glucan compared to high molecular weight β-glucan. These conditions should provide a greater opportunity for enzyme to degrade the diets which promotes the higher enzyme activities in gastrointestinal contents. Unfortunately, the modification of enzyme activity in duodenum and caecum was not enough to improve apparent digestibility in this study. The microbial population and community should be identified to understand and confirm this hypothesis.

### Health parameters

The relative weight of lymphoid organs (thymus and bursa of fabricius) was changed relating to immune response against antigen [14]. Bacterial cell wall contained β-glucan binding protein which can bind to immune cells and trigger immune responses [3]. An increase in spleen weight was observed in treatment groups receiving β-glucan [6,24], however in this study the spleen weight was not affected by the supplements. On the other hand, Rathgeber et al [12] did not find an influence on lymphatic organ weight from yeast β-glucan addition which may due to the low concentration (40 ppm) that they used compared to the study (200 and 400 ppm) of Guo et al [6]. The increase on relative weight of these immune organs was observed in this study, although a supplementation level is low (10 and 30 ppm) which may due to the using of low molecular weight β-glucan having a higher efficiency than the high molecular weight β-glucan used in the study of Rathgeber et al [12]. Therefore, appropriate molecular weight and concentration (10 or 30 ppm) were important factors in activating the immune response.

An improvement of phagocytic activities of macrophages and their function were observed in several studies [6,7] and in this study. The phagocytic activities of broilers were increased by the supplementation at 120 or 250 ppm, whereas a higher concentration at 500 ppm did not [7]. Moreover, higher phagocytic activities were observed in broilers fed diets with L but not for H. The high molecular weight may lack an effect on immune enhancement compared to low molecular weight when using fungal β-glucan. In contrast, the use of yeast β-glucan with molecular weight around 800 kDa had great efficiency enhancing immunity in broilers [6] which was similar in molecular weight to H in this study. Therefore, the different structural conformation of β-glucan between yeast and mold at high molecular weight could be the cause of these diverse outcomes, however further studies should be done to confirm this hypothesis.

Higher survival rate, lower pathogen accumulation in organs and better immunity parameters were clearly observed in broilers fed diets with β-glucan before pathogen challenges comparing to the control challenge without supplements group [13,19,20]. The high level of supplementation at 500 or 1,000 ppm provided a higher antibody titer against the vaccination [25], however this and other studies did not find the same results [7,9,14] may due to the lower level of supplementation.

Haematology and blood chemistry can reveal the blood, bone marrow and organ function. The β-glucan supplementation did not change these parameters in this study and other studies [9,14,24,25]. The digestive tract histology of broilers fed with β-glucan from plants deteriorate [5], whereas β-glucan from yeast and mold in this study did not cause these adverse effects [15,20]. Interestingly, the higher duodenal villi height found in this study should be a benefit as it would provide a larger area for absorption. However, the improvement in apparent digestibilty was not presented in this study.

### Carcass traits and meat quality

Carcass traits were not influenced by β-glucan supplementation in most studies [9,24,25], even using β-glucan from plants [5] which was similar to results in this study. However, some carcass traits were affected by the supplements. The lower gastrointestinal weight due to the shorter duodenal length was represented in this study, and was similar to other studies using yeast β-glucan [9]. The development of intestine correlated to bacterial community present, therefore, the changing of the bacterial community after β-glucan should be the cause. However, the further study should be performed to confirm this hypothesis.

In most studies, β-glucan did not influence meat colour except the study of Zhang et al [24] who reported a lower yellowness in breast meat after treatment by β-glucan which may due to the lower of abdominal fat. The lower brightness of breast meat was observed in the H group. The water composition in meat correlates to the meat brightness, however water composition and water holding capacity were not different between treatment groups. Therefore, the cause of this outcome should be confirmed in further study. In this study, the treatment did not effect on pH24, drip loss and chemical composition in meat which the same as others [18,24]. Therefore, the supplement of β-glucan did not have any serious adverse outcome on carcass traits and meat quality. In conclusion, the supplementation of L at 30 ppm in broiler chickens’ diet is recommended to obtain immune-modulating properties without adverse effects on productive performances, health, carcass traits and meat quality.

## Figures and Tables

**Table 1:** Ingredients and chemical composition of the basal diet

Items	Broiler diet	

	1 to 21 d	22 to 42 d
Ingredients (g/kg)		
Corn meal	517.2	621.8
Soybean meal	384.1	283.8
Soybean oils	53.9	54.8
Monodicalcium phosphate	21.1	17.3
Limestone	9.7	8.4
Sodium bicarbonate	2.0	2.0
Salt	2.4	2.4
Vitamin and mineral premix1)	2.0	2.0
L-lysine	1.2	1.5
DL-methionine	2.8	2.4
L-threonine	0.4	0.3
Choline chloride	0.7	0.8
Pellet binder	2.0	2.0
Maduramycin (Coccidiostat)	0.5	0.5
Calculated composition		
Metabolized energy (kcal/kg)	3,081	3,215
Analysed composition		
Dry matter (% fresh matter)	88.2	87.6
Crude protein (% dry matter)	21.8	18.1
Crude fat (% dry matter)	8.0	8.8
Crude fiber (% dry matter)	3.4	3.0
Crude ash (% dry matter)	6.4	5.4

1) Vitamin and mineral premix (Final B Prisma, IZA SRL) were supplied per kilogram of diets at 2,500,000 IU of vitamin A; 1,000,000 IU of vitamin D_3_; 7,000 IU of vitamin E; 700 mg of vitamin K; 400 mg of vitamin B_1_; 800 mg of vitamin B_2_; 400 mg of vitamin B_6_; 4 mg of vitamin B_12_; 30 mg of biotin; 3,111 mg of Ca pantothenate acid; 100 mg of folic acid; 15,000 mg of vitamin C; 5,600 mg of vitamin B_3_; 10,500 mg of Zn, 10,920 mg of Fe; 9,960 mg of Mn; 3,850 mg of Cu; 137 mg of I; 70 mg of Se.

**Table 2:** Productive performances of broilers between groups

Items	MW			Dose (ppm)				SEM	p-value		
		
	C	L	H	C	10	30	60		MW	Dose	MW×dose
Live weight (g)											
1 d	41.1	41.2	41.4	41.1	41.3	41.1	41.5	0.08	0.98	0.99	0.96
14 d	411	393	403	411	399	402	394	2.38	0.14	0.90	0.67
28 d	896	966	935	896	959	952	942	10.3	0.56	0.87	0.67
42 d	2,874	2,760	2,711	2,874	2,731	2,750	2,726	36.7	0.95	0.78	0.13
Average daily weigh gain (g)											
1–14 d	26.4	25.2	25.8	26.4	25.5	25.8	25.2	0.16	0.14	0.86	0.67
14–28 d	82.7	78.8	80.4	82.7	80.0	82.2	76.8	1.07	0.42	0.40	0.31
28–42 d	93.3	90.3	88.7	93.3	89.9	85.6	93.1	2.88	0.69	0.78	0.06
1–42 d	64.9	62.7	60.6	64.9	61.6	62	61.4	1.22	0.87	0.85	0.23
Average feed intake (g)											
1–14 d	31.4	32.2	30.3	31.4	31.7	31.5	30.6	1.04	0.61	0.89	0.71
14–28 d	113	111	111	113	113	114	107	2.01	0.78	0.43	0.26
28–42 d	157	152	143	157	147	151	146	1.87	0.20	0.54	0.28
1–42 d	101	98.2	95.0	101	97.3	98.3	94.3	0.80	0.34	0.20	0.99
Feed conversion ratio											
1–14 d	1.26	1.30	1.20	1.26	1.25	1.25	1.25	0.03	0.71	0.76	0.51
14–28 d	1.37	1.41	1.38	1.37	1.42	1.37	1.41	0.02	0.65	0.87	0.83
28–42 d	1.74	1.72	1.69	1.74	1.66	1.84	1.62	0.06	0.89	0.70	0.08
1–42 d	1.57	1.58	1.59	1.57	1.60	1.61	1.56	0.03	0.79	0.77	0.19

MW, molecular weight; C, control group; L, low MW β-glucan (8 kDa); H, high MW β-glucan (943 kDa); SEM, standard error of mean.

**Table 3:** Apparent digestibility and gut enzyme activities of broilers between the groups

Items	MW			Dose (ppm)				SEM	p-value		
		
	C	L	H	C	10	30	60		MW	Dose	MW×dose
Apparent digestibility (%)											
Dry matter	71.8	72.0	69.9	71.8	70.5	71.5	70.8	0.50	0.11	0.89	0.90
Organic matter	77.2	77.1	75.6	77.2	762	76.7	76.2	0.38	0.13	0.87	0.88
Crude protein	91.5	90.8	91.5	91.5	918	90.9	90.7	0.50	0.57	0.58	0.58
Ether extract	93.8	93.5	92.0	93.8	94.7	89.9	93.7	1.21	0.34	0.06	0.14
Enzyme activities in duodenal content											
Protease (U)	0.83	0.76	0.79	0.83	0.78	0.72	0.82	0.02	0.52	0.83	0.63
Amylase (U)	1.27^a^	1.54^b^	1.32^a^	1.27^a^	1.59^b^	1.6^b^	1.10^a^	0.02	0.04	0.03	0.59
Enzyme activities in caecum content											
Protease (U)	0.47	0.49	0.40	0.47	0.51	0.48	0.34	0.04	0.93	0.95	0.79
Amylase (U)	1.16^a^	1.81^c^	1.45^b^	1.16^a^	1.63^b^	1.56^b^	1.70^c^	0.03	0.03	0.05	0.83
Cellulase (U)	0.52	0.48	0.45	0.52	0.44	0.50	0.46	0.08	0.31	0.99	0.93

MW, molecular weight; C, control group; L, low MW β-glucan (8 kDa); H, high MW β-glucan (943 kDa); SEM, standard error of mean.

a–c Values within a row with different superscripts differ significantly at p<0.05.

**Table 4:** Organs, haematology, blood chemistry, phagocytic activity, antibody production and digestive tract histology of broilers between the groups

Items	MW			Dose (ppm)				SEM	p-value		
		
	C	L	H	C	10	30	60		MW	Dose	MW×dose
Organs at 25 days (% live weight)											
Pancreas	0.21	0.25	0.22	0.21	0.24	0.22	0.21	0.006	0.11	0.09	0.12
Spleen	0.07	0.09	0.08	0.07	0.07	0.09	0.07	0.002	0.28	0.45	0.60
Bursa of Fabricius	0.19^a^	0.24^b^	0.19^a^	0.19^a^	0.22^b^	0.23^b^	0.19^a^	0.007	0.03	0.05	0.63
Thymus	0.13^a^	0.21^b^	0.16^a^	0.13^a^	0.19^ab^	0.21^b^	0.13^a^	0.015	0.01	0.04	0.96
Haematology											
Haematocrit (%)	26.8	27.0	27.5	26.8	26.8	27.5	26.8	0.33	0.51	0.70	0.25
Erythrocytes (×10^6^/μL)	4.22	4.19	4.20	4.22	4.22	4.17	4.22	0.01	0.60	0.42	0.31
Leukocyte (×10^9^/L)	13.9	13.9	13.9	13.9	14.0	13.7	13.9	0.09	0.31	0.09	0.20
Blood chemistry											
Plasma protein (g %)	3.93	4.29	4.02	3.93	4.04	4.18	3.93	0.13	0.40	0.94	0.54
Blood urea nitrogen (mg/dL)	3.25	2.95	3.65	3.25	3.08	3.34	3.25	0.25	0.35	0.80	0.63
Creatinine (mg/dL)	0.16	0.20	0.20	0.16	0.20	0.21	0.16	0.01	0.99	0.61	0.97
Alanine aminotransferase (U/dL)	36.5	25.8	28.3	36.5	23.6	29.1	36.5	1.31	0.24	0.27	0.07
Cholesterol (mg/100 mL)	118	123	120	118	122	124	118	3.42	0.90	0.92	0.73
Triglyceride (mg/100 mL)	74.0	94.5	100	74.0	106	101	74.0	4.66	0.39	0.49	0.80
Phagocytic activity on the abdominal exudative cell (%)											
Non-opsonized sRBC	26.5^a^	32.0^b^	26.0^a^	26.5^a^	25.4^a^	31.3^b^	30.4^b^	0.86	0.04	0.001	0.07
Opsonized sRBC	33.7^a^	41.4^b^	36. 1^a^	33.7	36.5	39.9	39.8	0.76	0.001	0.08	0.61
Number of sheep red blood cells: phagocytic cell											
Non-opsonized sRBC	2.41	2.38	2.40	2.41	2.37	2.37	2.43	0.02	0.15	0.65	0.67
Opsonized sRBC	2.45	2.46	2.41	2.45	2.46	2.42	2.44	0.02	0.46	0.30	0.60
Antibody titer against NDV (log2)	4.00	3.80	3.33	4.00	3.50	3.90	3.30	0.26	0.75	0.92	0.43
Digestive tract histology (μm)											
Duodenal villi height	1,006^a^	1,175^b^	1,044^ab^	1,006	1,131	1,150	1,049	46.2	0.01	0.79	0.62
Duodenal crypt depth	152	163	174	152	166	171	169	5.78	0.57	0.54	0.20

MW, molecular weight; C, control group; L, low MW β-glucan (8 kDa); H, high MW β-glucan (943 kDa); SEM, standard error of mean; sRBC, Sheep red blood cells; NDV, Newcastle disease virus.

a,b Values within a row with different superscripts differ significantly at p<0.05.

**Table 5:** Carcass characteristics of broilers between the groups

Items	MW			Dose (ppm)				SEM	p-value		
		
	C	L	H	C	10	30	60		MW	Dose	MW×dose
Slaughter weight (g)	2,874	2,760	2,711	2,874	2,731	2,750	2,726	36.7	0.67	0.88	0.97
HCW (g)	2,296	2,194	2,141	2,296	2,172	2,138	2,193	30.2	0.59	0.40	0.38
Feet (% HCW)	4.54	4.60	4.43	4.54	4.72	4.38	4.46	0.08	0.07	0.97	0.12
Head (% HCW)	3.44	3.76	3.61	3.44	3.86	3.62	3.57	0.07	0.12	0.21	0.26
Spleen (% HCW)	0.12^b^	0.11^ab^	0.10^a^	0.12	0.12	0.10	0.11	0.004	0.04	0.88	0.84
Gastrointestinal tract (% HCW)	6.75^b^	6.76^b^	6.48^a^	6.75	6.89	6.60	6.38	0.12	0.02	0.21	0.46
CCW (g)	2,183	2,220	2,160	2,183	2,145	2,194	2,232	23.0	0.34	0.34	0.37
Dressing out (%)	98.6	98.5	98.4	98.6	98.6	98.4	98.5	0.51	0.28	0.29	0.29
Liver (% CCW)	1.76	1.82	1.94	1.76	1.96	1.77	1.92	0.05	0.50	0.48	0.67
Breast (% CCW)	27.9	26.6	26.8	27.9	25.2	27.7	27.2	0.43	0.41	0.57	0.17
Thighs (% CCW)	26.5	27.3	27.3	26.5	26.5	28.1	27.4	0.44	0.72	0.15	0.10
Wings (% CCW)	9.61	9.35	9.71	9.61	9.72	9.42	9.45	0.17	0.82	0.42	0.86
Meat/bone ratio (%)	80.5	80.9	81.1	80.5	81.5	81.1	80.6	0.34	0.10	0.37	0.25

MW, molecular weight; C, control group; L, low MW β-glucan (8 kDa); H, high MW β-glucan (943 kDa); SEM, standard error of mean; HCW, hot carcass weight; CCW, cold carcass weight.

a,b Values within a row with different superscripts differ significantly at p<0.05.

**Table 6:** Meat quality of broilers between the groups

Items	MW			Dose (ppm)				SEM	p-value		
		
	C	L	H	C	10	30	60		MW	Dose	MW×dose
Meat colour (*Pectoralis* muscle)											
Lightness (L*)	58.9	58.9	58.5	58.9	58.8	59.2	58.3	0.28	0.31	0.41	0.56
Redness (a*)	2.02	2.26	2.26	2.02	2.16	2.26	2.37	0.14	0.07	0.65	0.25
Yellowness (b*)	8.23	9.19	8.95	8.23	9.29	9.45	8.48	0.25	0.23	0.83	0.51
Chroma (C*)	8.53	9.53	9.29	8.53	9.63	9.74	8.87	0.26	0.16	0.88	0.39
Hue (H*)	76.9	76.0	75.7	76.9	77.2	76.2	74.3	0.81	0.13	0.47	0.42
Meat colour (*Gastrocnemius* muscle)											
Lightness (L*)	53.8^b^	53.4^b^	52.0^a^	53.8	52.4	53.2	52.6	0.43	0.02	0.96	0.69
Redness (a*)	4.62	5.19	5.36	4.62	5.58	4.79	5.46	0.22	0.07	0.83	0.26
Yellowness (b*)	8.19	8.00	8.21	8.19	8.12	8.18	8.02	0.24	0.24	0.23	0.88
Chroma (C*)	9.60	9.68	9.94	9.60	10.0	9.56	9.86	0.25	0.09	0.50	0.84
Hue (H*)	60.7	57.2	56.4	60.7	55.6	59.1	55.7	1.17	0.17	0.25	0.22
Meat pH after 24 hours	5.97	5.99	5.98	5.97	5.96	5.99	6.01	0.02	0.19	0.72	0.87
Drip loss 24 hours (%)	1.79	2.04	1.70	1.79	1.18	2.08	2.35	0.18	0.88	0.63	0.29
Drip loss 48 hours (%)	2.27	2.32	2.35	2.27	2.10	2.43	2.48	0.09	0.63	0.60	0.58
Thio-barbituric acid reactive substance after storage at 4°C (μg malondialdehyde/kg) at											
1 d	501	498	458	501	433	536	465	21.6	0.17	0.31	0.51
5 d	8,90^b^	464^a^	736^b^	890	720	475	605	61.7	0.001	0.12	0.24
7 d	1,128	1,320	1,377	1,128	1,329	1,296	1,420	49.2	0.69	0.07	0.37
15 d	2,645	2,298	2,536	2,645	2,642	2,417	2,193	70.8	0.47	0.11	0.33
30 d	4,324	4,789	4,435	4,324	4,936	4,290	4,611	385	0.93	0.78	0.24
Chemical composition of *Pectoralis* muscle (% fresh matter)											
Moisture	76.0	74.6	74.0	76.0	74.2	75.1	73.7	0.40	0.45	0.26	0.49
Crude ash	1.21	1.24	1.23	1.21	1.30	1.17	1.24	0.03	0.98	0.99	0.47
Crude protein	21.5	22.7	23.1	21.5	23.8	22.1	22.8	0.36	0.49	0.96	0.40
Ether extract	1.94	2.22	1.99	1.94	1.72	2.14	2.46	0.16	0.72	0.32	0.97
Chemical composition of *Gastrocnemius* muscle (% fresh matter)											
Moisture	77.8	77.0	77.7	77.8	76.7	77.4	78.1	0.24	0.84	0.21	0.18
Crude ash	1.06	1.11	1.07	1.06	1.16	1.11	1.01	0.03	0.95	0.19	0.53
Crude protein	17.2	19.8	18.8	17.2	19.7	19.5	18.7	0.28	0.64	0.25	0.21
Ether extract	2.47	2.53	2.25	2.47	2.17	2.48	2.54	0.12	0.93	0.61	0.49

MW, molecular weight; C, control group; L, low MW β-glucan (8 kDa); H, high MW β-glucan (943 kDa); SEM, standard error of mean.

a,b Values within a row with different superscripts differ significantly at p<0.05.
